# Eyelid and scleral thermal injury following phacoemulsification in silicone oil: a case report

**DOI:** 10.1186/s12886-022-02646-x

**Published:** 2022-11-04

**Authors:** Yu-Kuei Lee, Szu-Han Chen, Jia-Horung Hung

**Affiliations:** 1 Department of Ophthalmology, National Cheng Kung University Hospital, College of Medicine, National Cheng Kung University, Tainan, Taiwan; 2 Division of Plastic and Reconstructive Surgery, Department of Surgery, National Cheng Kung University Hospital, College of Medicine, National Cheng Kung University, Tainan, Taiwan; 3 Institute of Clinical Medicine, College of Medicine, National Cheng Kung University, Tainan, Taiwan

**Keywords:** Phacoemulsification, Fragmatome, Silicone oil, Thermal burn, Case report

## Abstract

**Background:**

Phacoemulsification has been the mainstay method for extracapsular cataract extraction surgery in the anterior segment; for cases of posterior drop of lens fragments into the vitreous, a posterior segment phacoemulsification instrument (fragmatome; Alcon, Inc., Fort Worth, TX) can be employed to remove the dislocated lens materials. Studies have reported on thermal injury to the cornea during phacmoemulsification of the anterior segment. However, few studies have investigated thermal burn in the simultaneous sclera and eyelid induced by the fragmatome. Currently, there is no reports and lack of optimal strategy for the management of nucleus drop in a vitreous cavity filled with silicon oil.

**Case Presentation:**

We present the case of a 53-year-old male patient with a thermal burn wound on the upper eyelid and sclera following phacoemulsification for a dropped lens in a silicone oil-filled vitreous. We further designed an experiment to verify our hypothesis that thermal injury could be induced by the high temperature of the metal tip during phacoemulsification in silicone oil. In our experiment, during 420 s of continuous ultrasonic wave, the temperature of the fragmatome tip in the balanced salt solution (BSS) increased from 22.0 to 24.0 ºC, while the temperature of the fragmatome tip in the silicone oil group increased from 22.0 to 43.0 ºC.

**Conclusions:**

The temperature of the fragmatome tip increased significantly in silicone oil compared to BSS in the experiment. Thus, physicians should be aware of possible thermal complications when using fragmatome in eyes filled with silicone oil.

## Background

Phacoemulsification is the mainstay method for extracapsular cataract extraction surgery owing to its favorable outcomes and relatively low incidence of complications [1, 2]. For cases with posterior drop of lens fragments into the vitreous, a posterior segment phacoemulsification instrument (fragmatome; Alcon, Inc., Fort Worth, TX) can be employed along with a 20-gauge vitrectomy system (Accurus®; Alcon Laboratories) to eliminate the dropped lens materials [3]. Ultrasound phacoemulsification utilizes piezoelectric crystals for creating rapid mechanical vibration, which emulsifies the lens but simultaneously generates heat, which can cause thermal injury to the cornea [4–6]. The improvement of the modern phacoemulsification instrumentation, specifically the invention of torsional phacoemulsification [7], can reduce thermal energy generation and decrease the temperature rise of the phacoemulsification tip during surgery [8]. However, since the fragmatome delivers energy via longitudinal phacoemulsification (40 kHz) [3], the risk of significant temperature rise during the surgery still exists, especially in the absence of fluid flow in a silicone oil-filled eye. Currently, there is no reports and lack of optimal strategy for the management of nucleus drop in a vitreous cavity filled with silicon oil and optimal strategy for the management of such complications is still unknown. Herein, we report a case of thermal injury to the eyelid and sclera following the use of the fragmatome to eliminate dislocated lens materials. Furthermore, we utilized an infrared thermometer to verify our hypothesis that thermal injury could be induced by the high temperature of the metal tip during phacoemulsification in silicone oil.

### Case presentation

**Fig. 1:**
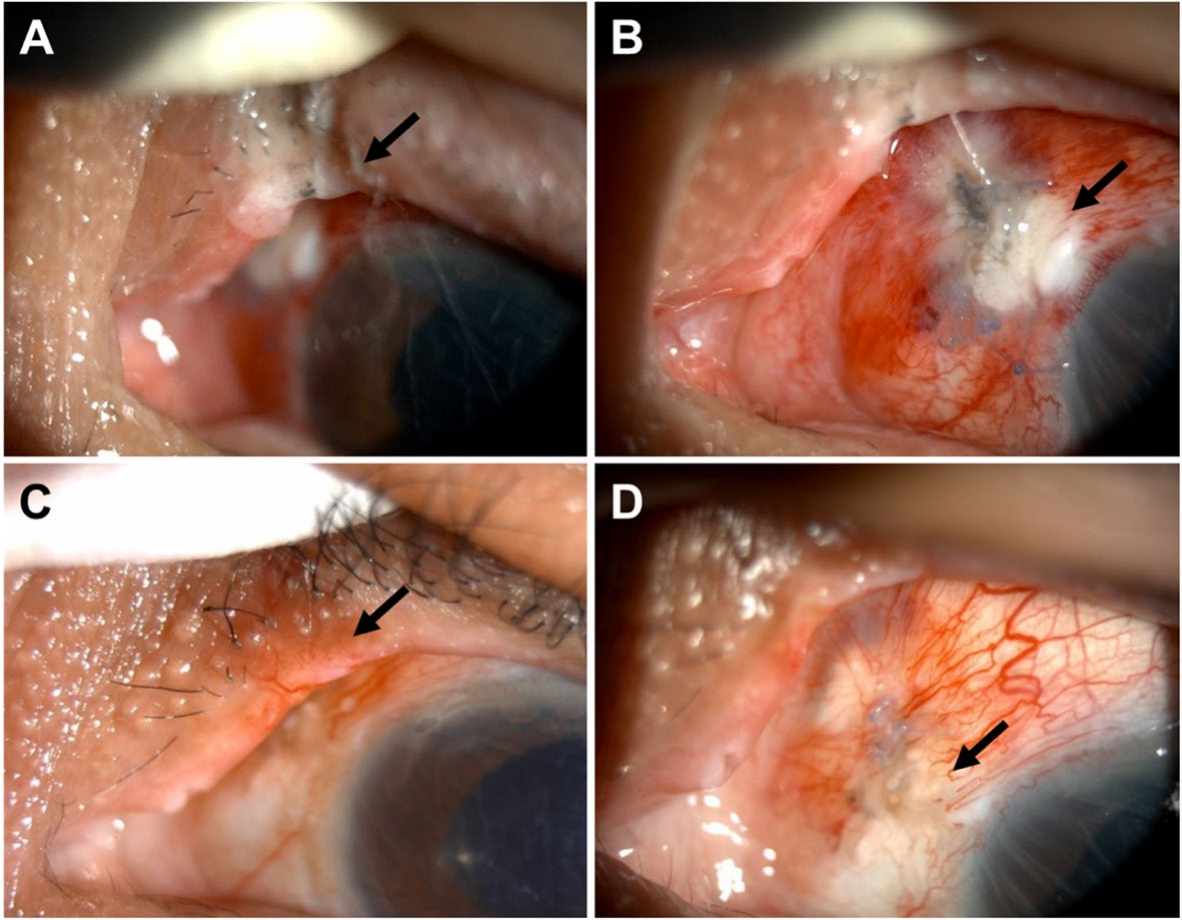
**(A)** A whitish wound (arrow) with scorch and tissue defect on nasal aspects of left upper eyelid were noted; (**B**) The sclerotomy site (arrow) also revealed a focal avascular area; **(C)** One month later, the upper eyelid wound (arrow) healed with scar formation; **(D)** The sclerotomy site (arrow) healed with scar formation

A 53-year-old man with a history of hepatitis B virus infection was admitted to our hospital owing to sudden vision loss in his left eye. On ocular examination, the best-corrected visual acuity was 20/30 in the right eye and hand motion at 60 cm in the left eye. Fundoscopic examination demonstrated near-total rhegmatogenous retinal detachment with breaks at the 4 and 7 o’clock positions in the left eye. The patient underwent placement of an encircling scleral silicone sponge (#506G; MIRA Inc.) and pars plana vitrectomy with silicone oil tamponade An incidental lens contact with posterior capsular damage was noted intraoperatively. Cataract gradually matured and obscured the fundal view; thus, phacoemulsification was performed two weeks later. During the operation, a fragmatome was used through the sclerotomy to remove the dropped lens with suction pressure of 400 mm Hg, flow limit 20 ml/min and an ultrasonic power output of 70% with pulse rate 5 pulse/second. The followability of the lens was poor in silicone oil and lens material was also difficult to be grasped and pulled out. Lens materials were usually pushed into the peripheral vitreous cavity by the fragmatome tip. Because the room lighting and illumination of the microscope were turned off and both the surgeon and the assistant concentrated on the fundal view under the microscope, so change of the eyelid or sclera were ignored. After the dropped nuclear materials were removed and illumination of the microscope and room lighting were turned on, a whitish wound with scorch and tissue defect on the nasal aspect of the left upper eyelid were noted (Fig. 1A, arrow). Examination of the superonasal sclerotomy site, which was created for the passage of fragmatome, also revealed a focal avascular area that resemble the scleral tissue following electro-cauterization. (Fig. 1B, arrow). An area of focal greyish necrosis was noted surrounding the whitish avascular area. Afterwards, the sclerotomy was closed with 7–0 vicryl sutures, and there was no postoperative leakage. We prescribed tobramycin and dexamethasone ointment (Tobradex®, Alcon, Rijksweg, Belgium) twice a day for the suspected thermal wounds on the eyelid and sclera. Because of the posterior capsular tear, the Sensar® 3-piece Intraocular Lens (AR40E, Johnson & Johnson, CA, US) was inserted and placed into the ciliary sulcus. The retina was attached under silicone oil tamponade. After following-up for one month, both eyelid and scleral wounds healed gradually with scar formation (Fig. 1C & D). The patient was then lost to follow-up.

## Experiment setup

**Fig. 2:**
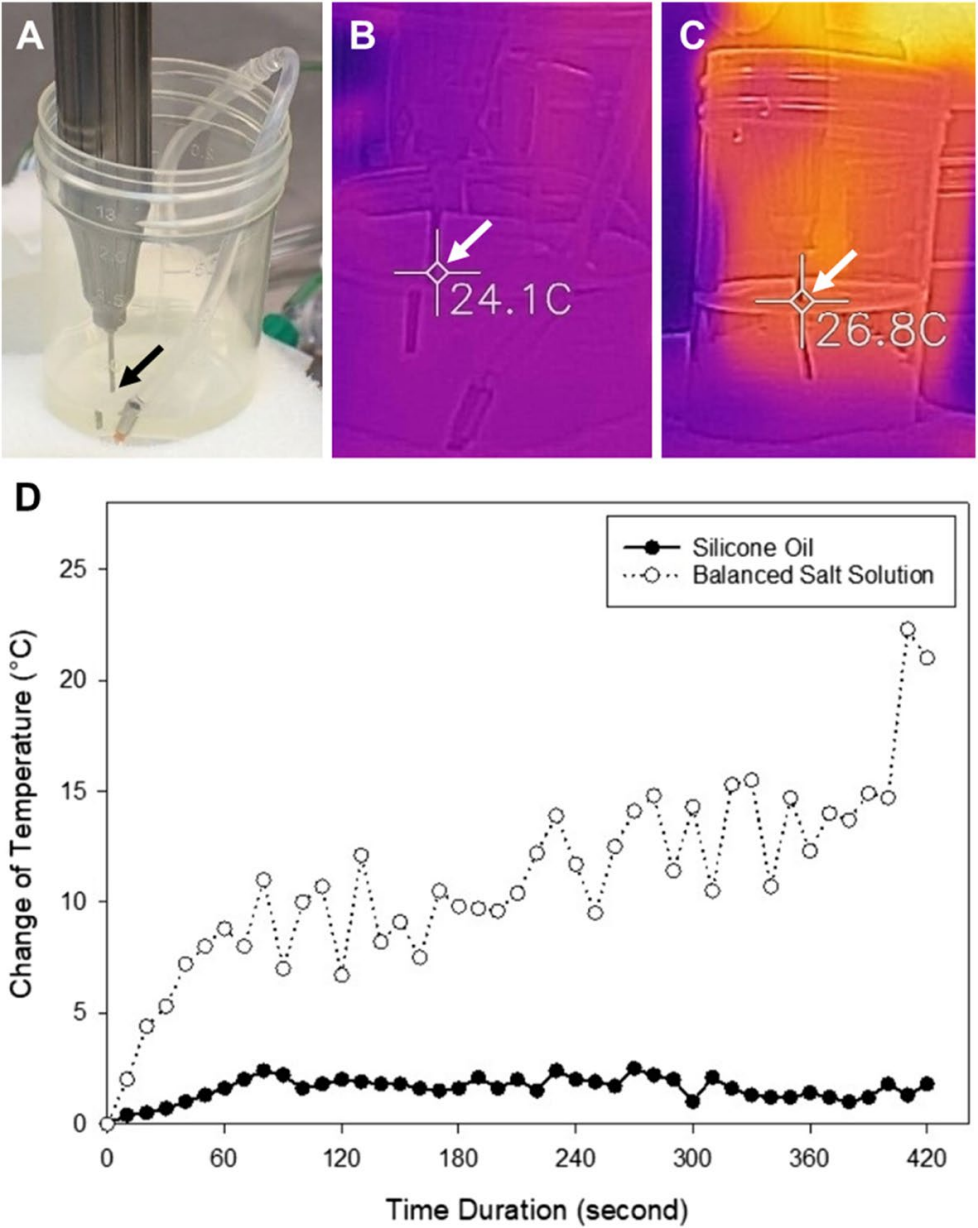
**(A)** The fragmatome tip was inserted 1 cm beneath the fluid level (arrow); **(B)** A high-sensitivity infrared thermal camera was used to detect the temperature of the fragmatome tip at the fluid level (arrow) of the balanced salt solution. **(C)** The thermal imager revealed the temperature of the fragmatome tip at the fluid level (arrow) of the silicone oil. **(D)** The change in the temperature at the fragmatome tip in the balanced salt solution and silicone oil in 240 s

The Alcon Constellation® Vision System was used as an ultrasonic emulsification aspiration device, and a 20-gauge Fragmatome Accessory Pak (Alcon Laboratories) ultrasonic human-lens fragmentation tip was used as the ultrasonic tip. A specimen collection bottle (60 mL) was filled with 15 mL of the test liquid. We inserted a fragmatome tip 1 cm beneath the liquid level (Fig. 2A). To measure the temperature of the fragmatome tip, a high-sensitivity infrared thermal camera system (FLIR ONE Gen 3, FLIR® Systems, Inc.) was used as a thermometer 3 cm away from the fragmatome tip, and the image focused on the junction of the fragmatome tip and the fluid surface (Fig. 2B and C). The experiment was performed in an operating room where the temperature was set at 22.0 ºC. For the experiment, phacoemulsification was setup under a suction pressure of 400 mm Hg, flow limit 20 ml/min and an ultrasonic power output of 70% with pulse rate 5 pulse/second.

## Test setup

### Set 1: Balanced salt solutions

For the balanced salt solution (BSS) test, a 23-G infusion cannula was placed into the specimen collection bottle filled with 15 mL BSS. After the BSS was released from the infusion line, the fragmatome tip was inserted into the BSS. A continuous ultrasonic wave was then generated for 420 s using the experiment settings. The change in temperature was recorded with a thermal imaging infrared camera every 10 s.

### Set 2: Silicone oil

For the silicone oil test, 15 mL of silicone oil (FCI, purified silicone oil, 5000 cSt, Syringe 10 mL) was injected into the specimen collection bottle. Continuous ultrasonic wave and temperature measurements were set up similar to those in Set 1.

### Outcomes

The temperature of the fragmatome tip in the BSS increased from 22.0 to 24.0 ºC (+ 2.0 ºC) at the 70^th^ s and then moved up and down in a range of 0.5ºC till 420^th^ s. The temperature of the fragmatome tip in the silicone oil group increased rapidly from 22.0 to 43.0 ºC (+ 21.0 ºC) at the 420^th^ s, with the highest temperature of 44.3 ºC (+ 22.3 ºC) at the 410^th^ s (Fig. 2D).

### Discussion and Conclusion

We present a case of a thermal burn wound on the upper eyelid and sclera following the use of a fragmatome in phacoemulsification surgery. Our hypothesis was that the heat could rapidly accumulate around the fragmatome tip when operated in silicone oil when there is nearly an absence of the silicone oil flow. We designed an experiment to verify our hypothesis. The results demonstrated that the temperature of the fragmatome tip significantly increased in silicone oil compared to that in BSS.

Ultrasound phacoemulsification transducers convert electrical energy to mechanical energy to enable piezoelectric materials of the phaco tip to vibrate at frequencies between 28 and 40 kHz [6]. The heat generated within the corneal incision wound is affected by the flow rate through the phaco tip, flow surrounding the tip within the incision, handpiece frequency, and stroke length [9]. During phacoemulsification, the thermal energy around the tip may generate and accumulate heat, which increases the temperature.

After the phaco tip’s activation, the time delay between the onset of phaco power and the onset of irrigation flow, and the diminished or interrupted irrigation fluid caused a rise in the temperature at the corneal incision site [4, 5, 10]. Thermal damage to the incision wound site may result in corneoscleral wound leakage and adjacent corneal stroma and endothelial damage, which induce corneal endothelial loss [4, 11]. Although several studies have focused on thermal injury to the cornea during phacmoemulsification in the anterior segment, there are no studies investigating fragmatome-induced thermal burns in the sclera and eyelid.

Phacoemulsification in the silicone oil was inefficient and technically difficult. The viscosity of silicone oil was much higher than that of water, so the lens in the silicone oil was barely movable. In our case, phacoemulsification occurred in the vitreous that had already been filled with silicone oil. The high viscosity of silicone oil could cause the occlusion of the fragmatome tip, which leads to a closed-tip condition, and therefore, the temperature can increase and damage the scleral tissue [12]. Furthermore, the thermal conductivity of silicon oil (0.15 W/ m/ K) is roughly a quarter of that of water (0.61 W/m/K) at normal temperature and pressure [13, 14]. During phacoemulsification in silicone oil, because of the minimal aspiration flow, the followability of the lens was poorer when compared to phacoemulsification in BSS. Lens fragments were also difficult to be grasped and pulled out. Lens fragments in the silicone oil were usually pushed into the peripheral vitreous cavity because the longitudinal stroke of the fragmatome tip tended to push nuclear fragments away even as the aspiration attracted (also known as chattering). The surgeon should pay special attention not to damage the retina while attempting to embed, grasp and emulsify the lens material using the fragmatome tip. Therefore, these factors might make surgical time of phacoemulsification longer, which led to heat accumulation and wound burn. The thermal burn ensued when the fragmatome tip passed through the sclera and simultaneously touched the upper eyelid. Fortunately, the patient’s burn wound eventually healed. 

We presented a rare but avoidable complication of phacoemulsification of the dropped nucleus in the vitreous filled with silicone oil. Our proposed strategy to prevent burn injury of the sclera and eyelid include: (1) remove the silicone oil and replace it with BSS prior to phacoemulsification (2) If the retina is detached after the removal of silicone oil, use perfluorocarbon to float away the nucleus and stabilize the posterior pole.

This report presented a case with a thermal burn on the upper eyelid and sclera following phacoemulsification for a dropped lens in a silicone oil-filled vitreous. To the best of our knowledge, this rare complication has not been reported in the literature. The strength of this study also includes an experiment to prove our hypothesis. However, limitations of this study include a lack of advanced examination imaging (e.g. anterior segment optical coherence tomography), and a relatively short follow-up period. Additional observational studies with longer follow-up period are suggested to re-confirm the findings of current study.

In conclusion, we report a case of thermal burns of the eyelid and sclera following phacoemulsification in eyes filled with silicone oil. The thermal burn might have been caused by the high temperature of the fragmatome tip. Care should be taken when using the fragmatome in eyes filled with silicone oil.

## Data Availability

The datasets used and/or analyzed in the course of the current study are available from the corresponding author on reasonable request.

## Abbreviation

BSS: Balanced salt solution
