# Correction: Eyelid and scleral thermal injury following phacoemulsification in silicone oil: a case report

**DOI:** 10.1186/s12886-022-02706-2

**Published:** 2022-12-02

**Authors:** Yu-Kuei Lee, Szu-Han Chen, Jia-Horung Hung

**Affiliations:** 1 Department of Ophthalmology, National Cheng Kung University Hospital, College of Medicine, National Cheng Kung University, Tainan, Taiwan; 2 Division of Plastic and Reconstructive Surgery, Department of Surgery, National Cheng Kung University Hospital, College of Medicine, National Cheng Kung University, Tainan, Taiwan; 3 Institute of Clinical Medicine, College of Medicine, National Cheng Kung University, Tainan, Taiwan

**Correction: BMC Ophthalmol 22, 420 (2022)**

**https://doi.org/10.1186/s12886-022-02646-x**

Following publication of the original article [1], the author group has identified an error in Fig. 2. D. The correct figure is given below.

**Fig. 2:**
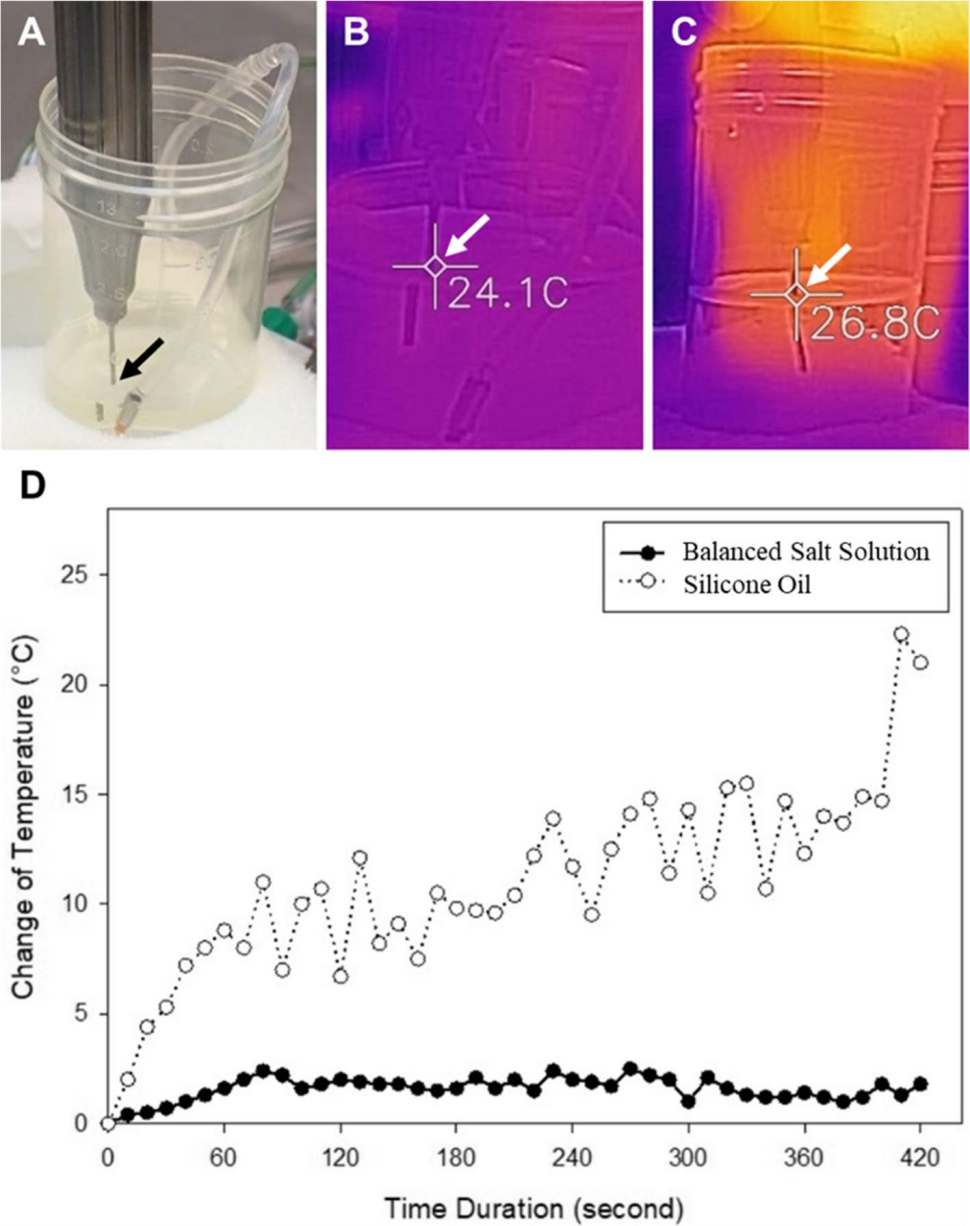
**A** The fragmatome tip was inserted 1 cm beneath the fluid level (arrow); **B** A high-sensitivity infrared thermal camera was used to detect the temperature of the fragmatome tip at the fluid level (arrow) of the balanced salt solution. **C** The thermal imager revealed the temperature of the fragmatome tip at the fluid level (arrow) of the silicone oil. **D** The change in the temperature at the fragmatome tip in the balanced salt solution and silicone oil in 240 s

The original article [1] has been corrected.
